# Interferon-Stimulated Gene-Mediated Defense Against Prion Infection: Noncanonical Function of Oas1a

**DOI:** 10.3390/v18090955

**Published:** 2026-09-01

**Authors:** Takujiro Homma, Daisuke Ishibashi

**Affiliations:** 1 Department of Pharmacology, Osaka Metropolitan University, Osaka 5458585, Japan; 2 Department of Immunological and Molecular Pharmacology, Fukuoka University, Fukuoka 8140180, Japan

**Keywords:** prion diseases, neurodegeneration, interferon (I-IFN), IRF3, Oas1a, innate immunity, prion protein conversion

## Abstract

Prion diseases are invariably fatal neurodegenerative disorders characterized by the conformational conversion of the host-encoded prion protein (PrP^C^) into its misfolded, pathogenic isoform (PrP^Sc^). Unlike conventional infectious agents such as viruses, prions lack nucleic acids, leading to the long-held assumption that they escape immune surveillance. Accumulating evidence, however, challenges this view and implicates the innate immune system in shaping prion pathogenesis. Yet, the underlying molecular mechanisms remain incompletely defined. Among innate immune pathways, our studies have implicated type I interferons (I-IFN), traditionally recognized as antiviral mediators, in the regulation of prion propagation and disease progression. We demonstrated that IRF3, a central transcriptional activator of I-IFN, restricts prion propagation and delays the onset of disease. Moreover, I-IFN itself enhances cellular resistance to prion infection in both in vitro and in vivo systems. Our recent work identified 2′-5′-oligoadenylate synthetase 1A (Oas1a), a downstream effector induced by I-IFN, as a mediator of noncanonical anti-prion activity. Genetic deletion of Oas1a increases susceptibility to prion infection, whereas recombinant Oas1a or enforced expression of Oas1a restores resistance. Oas1a blocks prion propagation by directly binding to PrP^C^ and interfering with its conversion into PrP^Sc^. This protective activity depends on Oas1a oligomerization, but not on its canonical 2′-5′-oligoadenylate synthetase activity. Collectively, these findings support a model in which the IRF3–I-IFN–Oas1a axis contributes to host defense against prion infection, at least in the experimental systems examined to date. In this review, we highlight current evidence for the involvement of this axis in innate immune responses to prion infection and discuss its mechanistic and potential therapeutic implications.

## 1. Introduction

Prion diseases are a group of transmissible, fatal neurodegenerative disorders caused by the conformational conversion of the normal cellular prion protein (PrP^C^) into a misfolded, pathogenic form (PrP^Sc^) [1]. This group includes scrapie in sheep, chronic wasting disease (CWD) in cervids, bovine spongiform encephalopathy (BSE) in cattle, and Creutzfeldt–Jakob disease (CJD) in humans. Pathogenic PrP^Sc^ self-propagates by converting native PrP^C^ into its misfolded isoform, leading to protein aggregation, neuronal dysfunction, and ultimately widespread neurodegeneration. Despite decades of intensive study, effective therapies remain elusive, and the molecular and immunological mechanisms driving prion pathogenesis remain incompletely understood.

Prion diseases are distinct among neurodegenerative disorders because of their transmissibility in the absence of nucleic acids, which historically fostered the assumption that they evade immune recognition. Pattern recognition receptors (PRRs) such as toll-like receptors (TLRs), RIG-I, and MDA5 detect conserved microbial components known as pathogen-associated molecular patterns (PAMPs), activating interferon regulatory factors (IRFs)—a family of transcription factors that includes IRF3 and IRF7 [2,3]. This activation triggers the production of type I interferons (I-IFN), including IFN-α and IFN-β, along with other cytokines of both pro- and anti-inflammatory function. Once secreted, I-IFN binds to its receptor (IFNAR) and stimulates a broad repertoire of interferon-stimulated genes (ISGs) [4]. These ISGs restrict pathogen propagation at multiple stages by blocking entry, degrading nucleic acids, suppressing protein synthesis, interfering with assembly and release, amplifying immune signaling, and promoting apoptosis of infected cells.

We previously demonstrated that IRF3 is a critical mediator of host defense against prion infection [5]. We also showed that I-IFN, induced downstream of IRF3 activation, suppresses prion propagation [6]. These findings prompted us to investigate the downstream effectors responsible for the anti-prion activity of I-IFN. Among ISGs, we recently identified 2′-5′-oligoadenylate synthetase 1A (Oas1a) as a key mediator of the I-IFN-dependent suppression of prion propagation [7]. Unlike its canonical antiviral activity through RNase L activation, Oas1a functions via a distinct, noncanonical mechanism in the context of prion infection. This discovery highlights an expanded role for Oas1a in innate immunity, extending its function beyond viral restriction to prion pathogenesis. Current evidence suggests that prion infection engages an IRF3-dependent innate immune pathway that culminates in the induction of ISGs with anti-prion activity. The following sections summarize the evidence supporting each step of this pathway.

## 2. Revising the Myth: Are Prions Truly Immunologically Silent?

Prions have historically been regarded as immunologically silent. This assumption stems from the observation that prion diseases typically lack overt inflammation during early and middle stages, with little infiltration of peripheral immune cells [8], and from the failure of conventional immune modulators or vaccination strategies to modify disease course. It has long been thought that the host immune system fails to recognize prions because PrP^Sc^ shares an identical amino acid sequence with the host-encoded PrP^C^ and, unlike viruses or bacteria, prions lack a genome. Nevertheless, accumulating evidence suggests that prion infection can engage innate immune pathways despite the absence of conventional pathogen-associated molecular patterns.

One of the most intriguing aspects of prion biology is the existence of multiple strains, characterized by distinct PrP^Sc^ conformations and associated with specific clinical and pathological phenotypes [9]. These strains vary in incubation period, lesion distribution, glycosylation profile, and sensitivity to host immune responses. Such strain-specific features are remarkably stable and are faithfully propagated upon serial passage in experimental animals and cell cultures. Notably, interference between prion strains has also been documented. In a well-characterized model, prior infection with the attenuated, slow-incubating CJD strain SY markedly suppressed subsequent infection with the virulent, fast-incubating FU strain [10]. Similar strain interference has been observed in prion-infected neuronal cultures [11], suggesting that cell-intrinsic mechanisms may contribute to this phenomenon. In virology, strain interference can be mediated by I-IFN responses triggered by host recognition of viral components. The analogous interference observed in prion models raises the possibility that innate immune pathways, potentially involving I-IFN signaling, may also contribute to this phenomenon.

Early studies in the 1960s reported that interferon treatment of scrapie-infected mice failed to provide therapeutic benefit, leading to the prevailing view that interferon-mediated immunity was unlikely to play a major role in prion pathogenesis [12,13,14]. At that time, interferons had not yet been classified into type I, II, and III subtypes, nor had type I-IFN genes such as IFN-α and IFN-β been cloned, limiting mechanistic insight. Subsequent investigations appeared to reinforce this perspective: IFNs were undetectable in the serum, spleen, or brain of scrapie-infected mice [15], and no induction of IFN-β mRNA was observed in the brains of CJD patients or scrapie-infected mice [16,17]. However, despite the absence of overt I-IFN production, several ISGs, including Mx1 and OAS-related genes, were found to be upregulated in the brains of CJD patients and prion-infected animals such as hamsters and mice [16,18,19,20]. Moreover, increased expression of IRFs, which are key transcriptional regulators of the I-IFN pathway, has been detected in microglia from CJD-affected human brains during early stages of disease [16]. These findings suggest that prion infection may induce a weak or highly localized I-IFN response—undetectable by conventional assays—yet sufficient to activate downstream ISG expression in specific CNS-resident cell types.

## 3. Innate Immune Signaling in Prion Pathogenesis

Accumulating evidence indicates that multiple components of the innate immune system, including TLR and interleukin signaling, influence prion disease progression. Mice carrying a nonfunctional Toll-like receptor 4 (TLR4) mutation in the intracellular signaling domain develop disease with significantly shorter incubation periods than wild-type counterparts across multiple prion strains and inoculum doses [21]. These findings suggest that TLR4 signaling plays a protective role during prion infection. The reduced responsiveness of macrophages from these mutants to fibrillogenic PrP peptides [PrP(106–126) and PrP(118–135)] further raises the possibility that structurally abnormal PrP may engage TLR4-dependent innate immune signaling [21]. However, because these experiments employed PrP^Sc^-mimetic fibrillogenic peptides rather than native infectious PrP^Sc^, whether PrP^Sc^ itself is directly recognized through TLR4 remains unresolved.

CD14, a co-receptor involved in TLR signaling, also influences prion disease progression. Mice deficient in CD14 exhibit delayed PrP^Sc^ accumulation, delayed disease onset, and prolonged survival compared with wild-type mice [22]. Interestingly, these changes are accompanied by increased microglial activation, particularly during the early stage of infection, together with increased expression of anti-inflammatory cytokines such as IL-10 [22].

Myeloid differentiation primary response 88 (MyD88), an adaptor protein downstream of most TLRs and IL-1 receptors, appears dispensable for prion disease progression following intracerebral infection. MyD88-deficient mice show disease progression comparable to that of wild-type mice after intracerebral inoculation [23]. However, following peripheral infection with a low prion dose, MyD88-deficient mice exhibit delayed disease onset, suggesting that MyD88 may contribute to peripheral neuroinvasion rather than CNS prion propagation.

Cytokines that regulate inflammatory responses also influence prion disease progression. Interleukin-10 (IL-10) is a potent immunosuppressive cytokine. IL-10–deficient mice show markedly shorter incubation periods after both intracerebral and peripheral inoculation compared with wild-type controls, indicating a protective role for IL-10 in prion pathogenesis [24]. In contrast, interleukin-1 (IL-1) signaling appears to promote pathogenesis. Mice lacking IL-1 receptor type I exhibit prolonged survival and reduced accumulation of PrP^Sc^ compared with wild-type animals, suggesting that IL-1–driven glial activation contributes to neurodegeneration [25]. Notably, IRF3 activation in microglia suppresses IL-1 expression while enhancing IL-10 and IL-1 receptor antagonist production [26], suggesting that IRF3 may shift microglial responses toward a less inflammatory state. Together, these findings demonstrate that innate immune responses can exert either protective or detrimental effects on prion pathogenesis, depending on the pathway and experimental context. Thus, the IRF3–I-IFN axis represents one component of a broader innate immune network whose effects may vary according to cell type, disease stage, and route of infection.

## 4. IRF3: A Gatekeeper of Innate Immunity Against Prion Infection

The differential effects of TLR4 [21] and MyD88 [23] deficiency on prion disease raise the possibility that MyD88-independent TLR signaling contributes to host defense. TLR4 can signal through the TRIF adaptor, which activates IRF3 via TBK1/IKKε, leading to I-IFN production [27]. The TRIF–IRF3 pathway thus represents a potential MyD88-independent pathway involved in the host response to prion infection. IRF3 is constitutively expressed in a broad range of cell types, including lymphocytes, glial cells, neurons, and neuroblastoma cell lines [28,29]. Neurons also express innate immunity-related genes and can produce I-IFN in response to viral infection [30]. The TLR3–IRF3 signaling axis has been implicated in host responses to neurotropic viral infections, including herpes simplex encephalitis and rabies [31,32]. To examine the role of IRF3 in prion disease, we intraperitoneally inoculated IRF3-deficient mice with multiple prion strains. IRF3-deficient mice exhibited significantly shorter incubation periods than wild-type controls for all prion strains tested [5]. In addition, PrP^Sc^ was detected earlier in the spleens of IRF3-deficient mice.

Although terminal neuropathology in the brain—including spongiform change and PrP^Sc^ deposition—was not markedly different between groups, these findings suggest that IRF3 delays disease progression, particularly during the early peripheral phase of infection. Consistent with a protective role for IRF3, in vitro studies showed that IRF3 activation inhibited PrP^Sc^ accumulation in persistently infected cell cultures, while IRF3 overexpression conferred resistance to de novo prion infection, supporting a cell-autonomous anti-prion function of IRF3.

Notably, *Irf3* gene expression was significantly downregulated in N2a-22L cells, a murine neuroblastoma line persistently infected with the 22L scrapie strain, compared with uninfected controls. We therefore investigated whether this reduction resulted from altered transcriptional regulation. Because the promoter region of the mouse *Irf3* gene had not been previously characterized, we performed a luciferase reporter assay and identified the upstream −119 bp region as a functional promoter [33].

**Figure 1:**
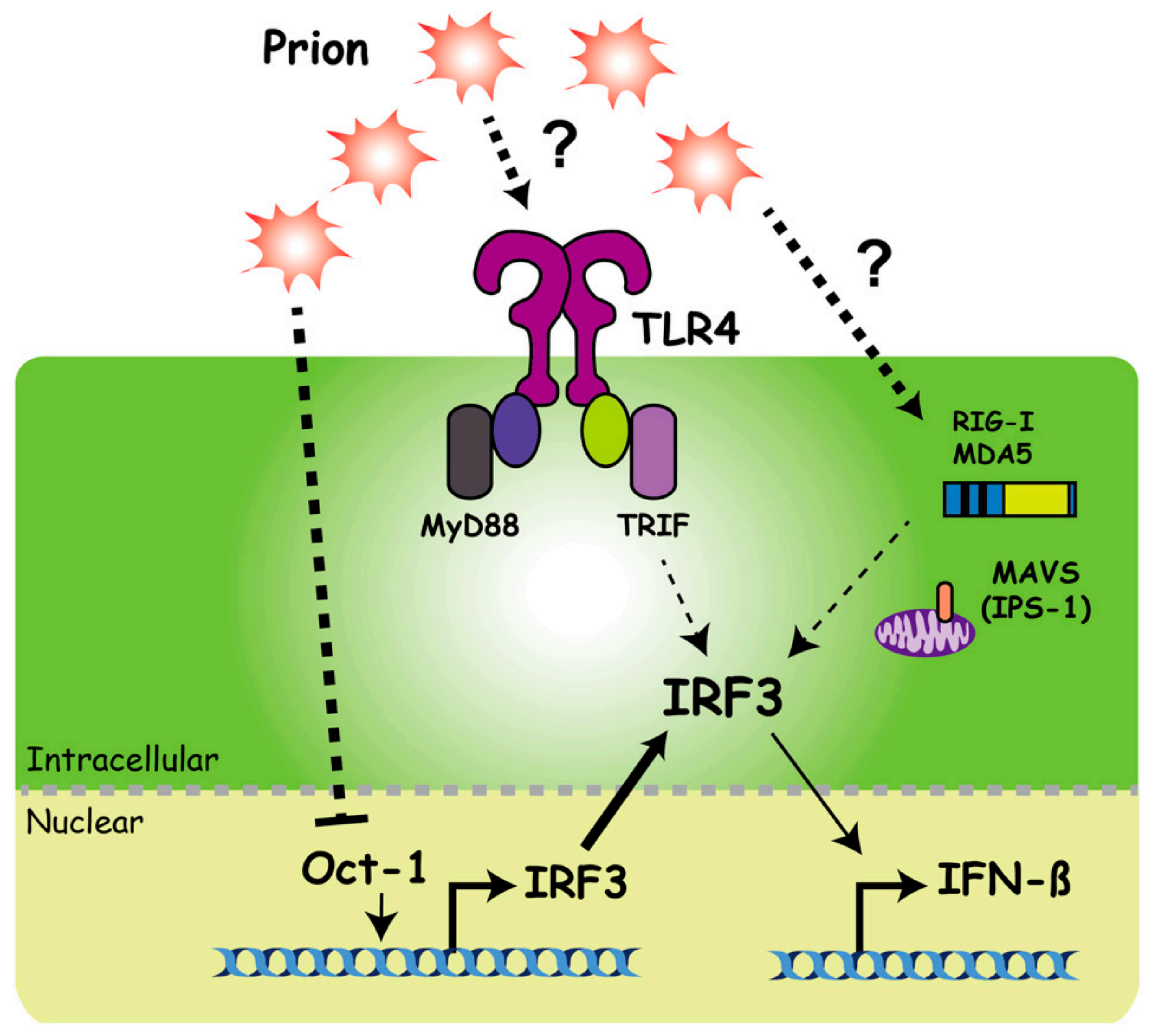
Proposed upstream mechanisms linking prion infection to IRF3 activation. The mechanisms by which prion infection engages IRF3 signaling remain unresolved. TLR4 represents one potential upstream pathway that can signal through TRIF to activate IRF3, although direct recognition of prions by TLR4 has not been established. RIG-I and MDA5 can also activate IRF3 through MAVS; however, because these receptors are nucleic acid sensors, there is currently no evidence that they directly recognize prions, and their potential involvement may instead reflect indirect activation by host-derived signals generated during prion infection. Activated IRF3 induces IFN-β expression. Prion infection may also suppress this pathway by reducing Oct-1 expression, thereby decreasing IRF3 transcription. Dashed arrows and question marks indicate proposed or unresolved links between prion infection and candidate upstream signaling pathways.

Accumulating evidence shows that many viruses have evolved mechanisms to evade host immunity, including IRF3-dependent signaling [34,35]. Several viruses promote IRF3 degradation or block its phosphorylation, thereby suppressing I-IFN production and enabling persistent infection [36,37,38,39,40]. *Irf3* promoter activity was markedly reduced in N2a-22L cells compared with uninfected controls. Strikingly, this suppression was reversed by treatment with pentosan polysulfate or Congo red, both well-established anti-prion compounds. Further analysis identified octamer-binding transcription factor-1 (Oct-1) as the principal regulator of *Irf3* promoter activity. Consistent with this, Oct-1 expression was significantly diminished in the brains of scrapie-infected mice and in N2a-22L cells. These findings indicate that prion infection is associated with reduced Oct-1 expression and impaired *Irf3* transcription, potentially weakening IRF3-mediated innate immune responses [33]. The observed inverse correlation between IRF3 expression and cellular susceptibility to prion infection further supports an anti-prion function for IRF3. However, it remains unclear how prion infection leads to Oct-1 downregulation and whether this effect is directly associated with PrP^Sc^ accumulation or results from secondary cellular changes induced by persistent infection (Figure 1).

## 5. I-IFN: Translating IRF3 Signals into Anti-Prion Action

IRF3 is a central transcription factor that drives the induction of I-IFN [41]. IRF3-deficient mice show impaired I-IFN production and increased susceptibility to viral infections [42,43]. Although robust I-IFN secretion is not consistently observed in prion-infected tissues or cells, many cell types display low-level constitutive I-IFN production [44]. Tonic I-IFN signaling may prime cells for subsequent stimuli and enhance their responsiveness. Recent findings indicate that tonic I-IFN signaling is not only important for amplifying immune responses upon pathogen challenge but also serves a cell-intrinsic function in preserving homeostasis and preventing malignant transformation [45].

**Figure 2:**
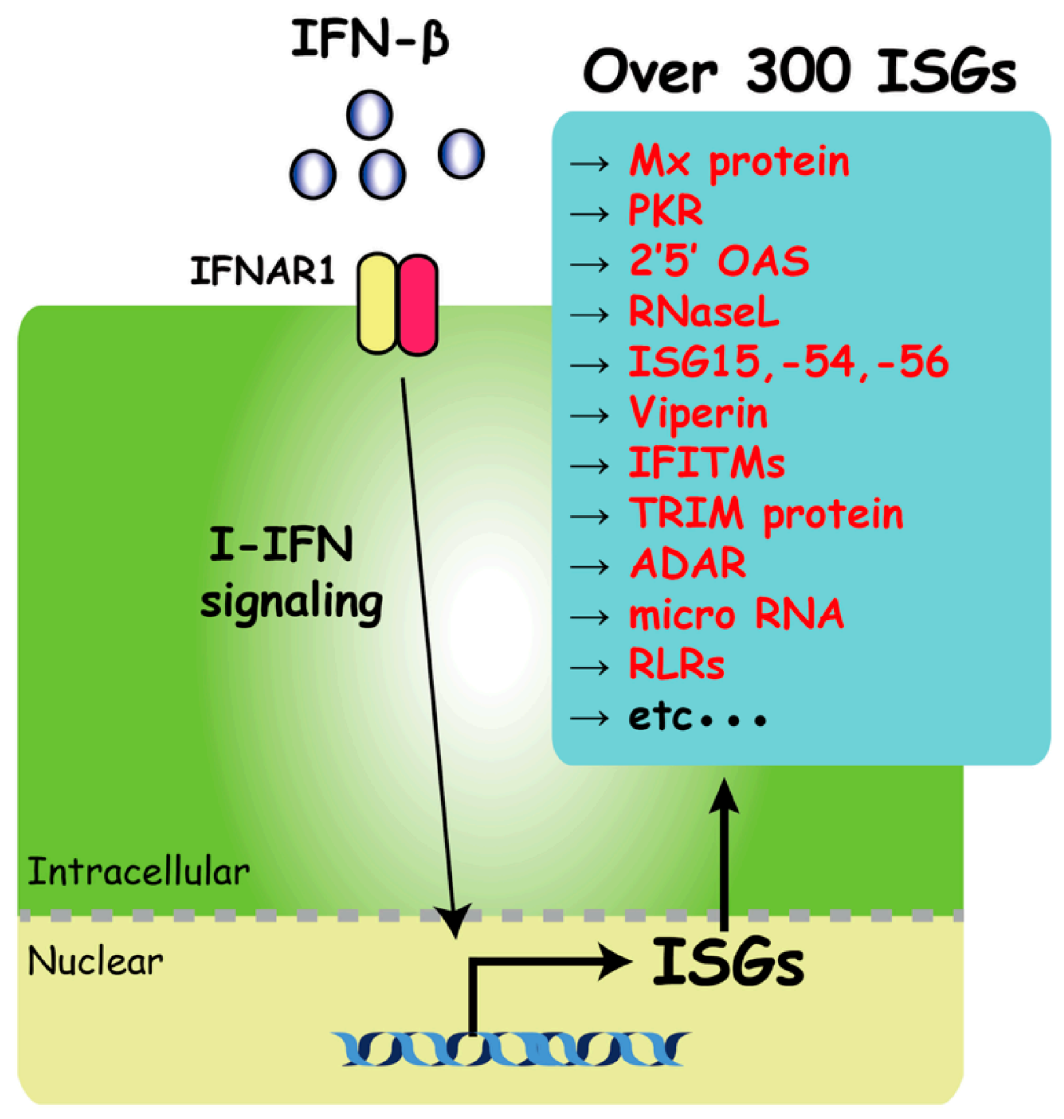
Type I interferon signaling and induction of interferon-stimulated genes. IFN-β binds to the type I interferon receptor (IFNAR) and activates intracellular I-IFN signaling, leading to the transcriptional induction of more than 300 interferon-stimulated genes (ISGs). Representative ISGs are shown.

I-IFNs, including IFN-α and IFN-β, signal through the IFNAR to activate the JAK-STAT pathway [46]. Following ligand engagement, STAT1 and STAT2 are phosphorylated and associate with IRF9 to form the interferon-stimulated gene factor 3 (ISGF3) complex, which translocates into the nucleus and induces ISG transcription (Figure 2). These ISGs restrict viral replication, regulate proliferation and apoptosis, and promote antigen presentation. Although direct evidence of I-IFN secretion in prion-infected brains remains limited, transcriptomic analyses have revealed upregulation of ISGs in both prion-infected animals and human CJD brain tissues [16,18,19,20]. In experimental models, these transcriptional changes are consistent with functional studies supporting a role for I-IFN signaling in restricting prion propagation. In human CJD, however, the available evidence is primarily transcriptomic, and the functional significance of these interferon-associated transcriptional changes remains to be determined.

To directly evaluate the role of I-IFN signaling in prion disease, we analyzed mice lacking IFNAR1, an essential subunit of IFNAR [46]. IFNAR1-deficient mice developed disease more rapidly, showing greater PrP^Sc^ accumulation in both brain and spleen and more severe neuropathology than wild-type controls [6]. Histological examination at 100 days post-inoculation—prior to clinical onset—revealed pronounced vacuolation, gliosis, and PrP^Sc^ deposition. In vitro, IFNAR1-deficient fibroblasts were more permissive to prion infection, a phenotype reversed by retroviral re-expression of IFNAR1. We also assessed the therapeutic potential of exogenous I-IFN. In prion-infected N2a cells, recombinant IFN-β markedly reduced PrP^Sc^ in a dose-dependent fashion. Similarly, the small-molecule IFNAR1 agonist RO8191 lowered PrP^Sc^ accumulation in vitro. In prion-infected mice, RO8191 treatment decreased PrP^Sc^ burden, gliosis, and vacuolation during the early and mid-stages of disease. The compound was detectable in both brain and spleen, indicating its ability to reach the CNS. Consistent with these outcomes, RO8191 treatment significantly extended survival in prion-infected mice [6]. Collectively, these experimental findings support a protective role for I-IFN signaling against prion infection and point to downstream ISGs as potential mediators of this effect.

## 6. Oas1a: An I-IFN–Induced Effector That Restricts Prion Conversion

IFN signaling induces over 300 ISGs [4], but which of these genes mediate anti-prion activity remains poorly defined. To identify downstream effectors, we tested the anti-prion activity of selected ISGs by monitoring the formation of newly synthesized protease-resistant PrP^Sc^ in a cell-based assay. Among the ISGs tested, Oas1a markedly reduced PrP^Sc^ formation without altering PrP^C^ expression or host protein synthesis, whereas other ISGs such as Mx1, ISG15, and Viperin showed no effect [7]. Oas1a, a member of the OAS family, is best known for its antiviral role through the synthesis of 2′-5′-linked oligoadenylates (2-5A), which activate RNase L to degrade viral RNA [47]. However, it also mediates RNase L–independent functions, including direct protein interactions and immunomodulatory activity [48,49,50]. To examine its physiological relevance, we generated Oas1a-deficient mice and inoculated them intracerebrally with the 22L prion strain. Compared with wild-type controls, Oas1a-deficient mice exhibited shorter incubation periods and significantly increased PrP^Sc^ accumulation in both brain and spleen. Histological analysis revealed enhanced PrP^Sc^ deposition along with pronounced microglial and astrocytic activation. These results support a protective role for Oas1a against prion propagation in vivo. Building on prior evidence that IFN-β inhibits prion propagation, we investigated whether this effect is mediated by Oas1a. In Oas1a-deficient cells, IFN-β failed to suppress prion propagation, whereas recombinant Oas1a restored resistance. In wild-type cells, recombinant Oas1a alone reduced prion propagation to a level comparable to that achieved with IFN-β. These findings indicate that Oas1a is required for IFN-β–mediated suppression of prion propagation and can itself exert anti-prion activity [7].

**Figure 3:**
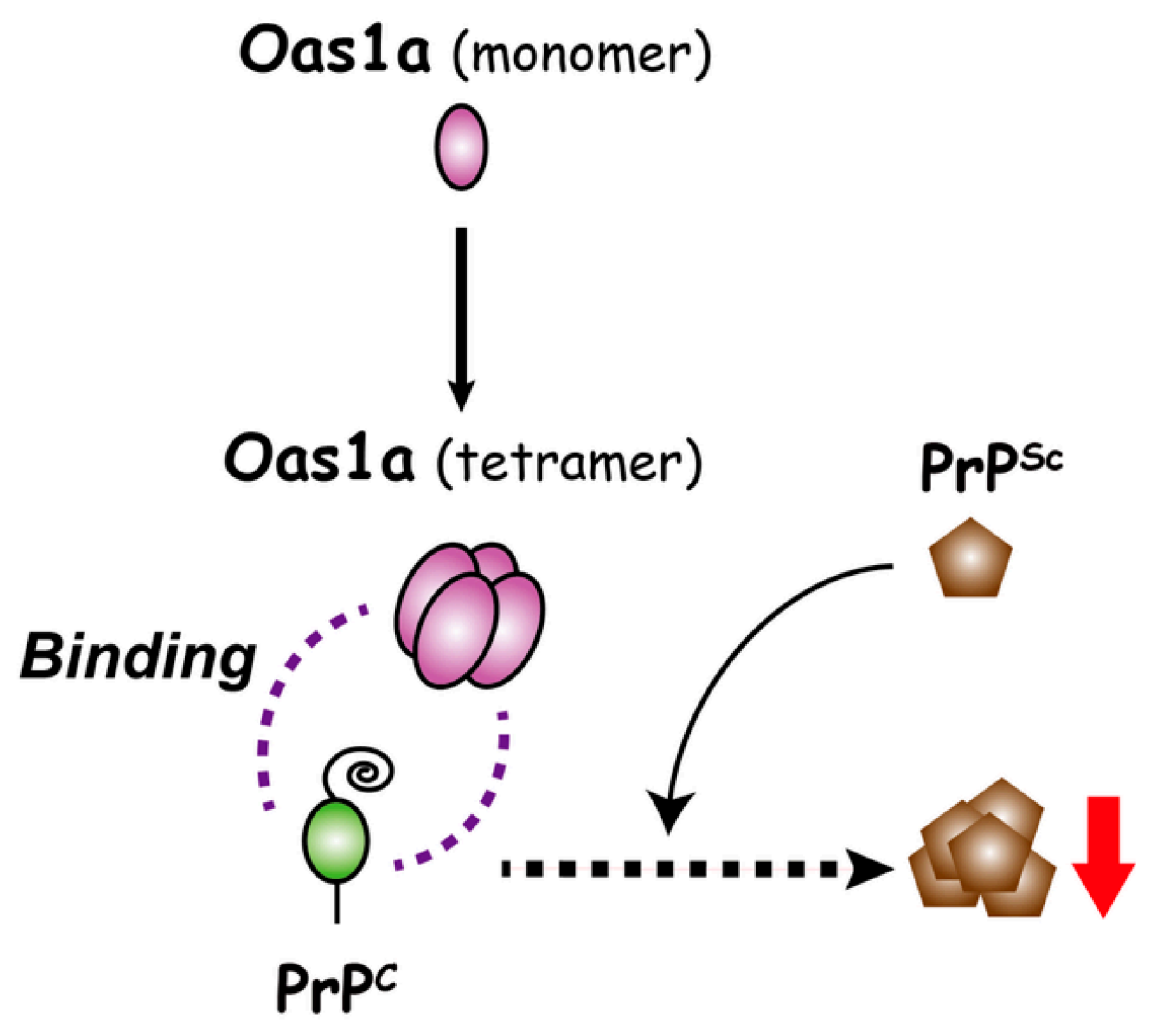
Noncanonical anti-prion mechanism of Oas1a. Oas1a suppresses prion propagation through a mechanism independent of its canonical enzymatic activity and RNase L activation. Oas1a tetramerization is required for its anti-prion activity. Tetrameric Oas1a binds to PrP^C^ and interferes with its pathological conversion into PrP^Sc^.

Biochemical and structural analyses further revealed that this function of Oas1a is independent of its canonical enzymatic activity. Tetramer formation—but not ATP binding or 2-5A synthesis—was required for the anti-prion effect, indicating a noncanonical mechanism. Surface plasmon resonance showed high-affinity binding of Oas1a to PrP^C^, while fluorescence microscopy demonstrated time-dependent colocalization of internalized Oas1a with PrP^C^, supporting a model in which Oas1a interacts with PrP^C^ and interferes with its pathological conversion (Figure 3). Together, these results identify Oas1a as a key effector of I-IFN–mediated anti-prion defense, acting through a noncanonical RNase L–independent mechanism involving direct interaction with PrP^C^. Increased expression of OAS-related genes has also been reported in prion-infected brains across multiple host species, including patients with CJD and experimentally infected mice and hamsters [16,19]. An important limitation, however, is that the direct anti-prion activity of Oas1a has thus far been demonstrated primarily in murine experimental systems using the 22L prion strain. Whether this noncanonical function of Oas1a is conserved across different prion strains and host species, and whether a similar OAS-mediated mechanism operates in human prion disease, remain to be determined.

## 7. Therapeutic Implications and Targeting the IRF3–IFN–Oas1a Axis

Experimental findings suggest that the IRF3–I-IFN–Oas1a axis may offer opportunities for therapeutic intervention in prion disease. Because Oas1a acts downstream of I-IFN signaling and suppresses prion propagation independently of RNase L, this pathway could be modulated at multiple levels, including interferon signaling, Oas1a expression, and Oas1a activity.

I-IFN Therapy: Early studies of interferon treatment in experimental prion disease did not demonstrate therapeutic benefit [12,13,14]. However, these studies were conducted before the molecular characterization of I-IFN signaling and its downstream ISGs, limiting mechanistic interpretation of the negative findings. Our subsequent findings demonstrated anti-prion effects of I-IFN in experimental models [6], providing a rationale for further investigation of this pathway. Recent advances in formulation, including pegylated IFNs and other long-acting variants, have improved interferon pharmacokinetics. Conjugation of polyethylene glycol (PEG) to interferon extends its half-life and permits less frequent dosing [51]. Pegylated interferons have been clinically used in several diseases, demonstrating that modification of interferon pharmacokinetics is feasible [52,53]. These developments warrant further experimental evaluation of whether modern interferon formulations can achieve anti-prion effects at tolerable levels, although their clinical applicability to prion disease remains uncertain.

Gene Therapy and Small Molecules: Gene-based approaches that provide sustained local expression of I-IFN in the CNS could be explored as a therapeutic approach. This concept is supported by our previous finding that intracerebral delivery of a lentiviral vector encoding IFN-β reduced PrP^Sc^ accumulation and neuropathological changes in prion-infected mice [6]. Defining how prions disrupt IRF3 signaling could reveal therapeutic opportunities to preserve this pathway, for example by preventing Oct-1 loss or maintaining IRF3 activity in infected neurons. Similarly, future studies could explore whether small molecules that promote Oas1a tetramer formation or enhance its interaction with PrP^C^ can increase its anti-prion activity. Combination strategies pairing I-IFN agonists or ISG inducers with therapies that lower PrP^C^ levels, such as antisense oligonucleotides, may also merit investigation.

Importance of Early Intervention and Translational Challenges: A major challenge in translating these findings into therapy is the long presymptomatic phase of prion diseases. By the time neurological symptoms become apparent, substantial PrP^Sc^ accumulation and neuronal damage may already have occurred, potentially limiting the therapeutic benefit of interventions aimed primarily at suppressing further prion propagation. Effective delivery to relevant CNS compartments and cell populations also represents an important challenge, particularly for biologic and gene-based approaches. In addition, the timing, magnitude, and duration of I-IFN signaling are likely to be critical determinants of therapeutic outcome. Although appropriately controlled I-IFN responses may restrict prion propagation, excessive or prolonged activation can promote neuroinflammation and other detrimental effects in the CNS, including microglial overactivation, synaptic dysfunction or loss, and disruption of neural homeostasis in neurodegenerative models [54,55,56]. Thus, therapeutic activation of I-IFN signaling will likely require careful control rather than sustained systemic stimulation. Future approaches will require optimization of treatment timing, dose, duration, and CNS delivery, potentially with cell- or tissue-targeted modulation of this pathway. These challenges will need to be addressed before the therapeutic potential of the IRF3–I-IFN–Oas1a axis can be translated beyond experimental models.

## 8. Key Concepts

1. Innate immune signaling may play a previously underappreciated role in prion pathogenesis. Experimental studies suggest that I-IFN-related pathways may influence prion propagation. Transcriptomic analyses have identified ISG upregulation in prion-infected animal and human CJD brain tissues. However, the functional significance of these transcriptional changes in human prion disease remains unclear.
2. IRF3 is an upstream regulator of MyD88-independent innate immune signaling that contributes to host defense against prion infection. IRF3 activation drives I-IFN production and downstream ISG induction. Its expression is diminished during chronic prion infection in association with Oct-1 downregulation. IRF3 deficiency accelerates disease progression in mice, whereas IRF3 activation or overexpression suppresses prion propagation in cell-based models, supporting its functional relevance in anti-prion defense.
3. Experimental studies support a protective role for I-IFN signaling in prion infection. Genetic disruption of IFNAR1 increases susceptibility to prion infection, whereas pharmacological activation of I-IFN signaling suppresses PrP^Sc^ accumulation and delays disease progression in experimental models. The extent to which these protective effects operate in human prion disease remains unknown.
4. Oas1a, a noncanonical ISG effector, suppresses prion propagation through a mechanism distinct from its classical antiviral role. Independently of 2-5A synthesis and RNase L activation, Oas1a binds directly to PrP^C^ and interferes with its pathological conversion. This anti-prion activity requires Oas1a tetramerization, suggesting a structural mode of action in restricting prion propagation.

## 9. Outstanding Questions Remain

1. What upstream mechanisms drive IRF3 suppression in prion-infected cells? Although Oct-1 downregulation has been implicated, the molecular link between prion infection and Oct-1 repression remains unresolved. It remains unclear whether suppression of the IRF3–I-IFN–Oas1a axis is induced by prion infection or whether reduced activity of this pathway precedes and facilitates prion propagation.
2. How does Oas1a interact with PrP^C^ to interfere with its pathological conversion? Although cell-based models support a direct interaction, the molecular basis of this binding remains unresolved, and in vivo validation is challenging. Future studies should determine whether Oas1a stabilizes native PrP^C^ or instead disrupts the conversion of PrP^C^ to PrP^Sc^ at membrane-associated sites.
3. How do host cells recognize prions in the absence of nucleic acids and classical PAMPs? One possibility is that abnormal PrP structures directly engage innate immune recognition pathways. Fibrillogenic PrP peptides have been shown to induce cytokine responses in macrophages via TLR4 [21], suggesting that altered PrP can stimulate innate immune signaling. However, these findings do not establish direct recognition of infectious PrP^Sc^ by TLR4. Alternatively, PrP^Sc^ accumulation may disrupt cellular homeostasis and indirectly activate innate immune responses through damage-associated mechanisms. Because RIG-I and MDA5 are nucleic acid sensors, there is currently no evidence that they directly recognize proteinaceous PrP^Sc^. If these pathways are involved, their activation would more likely occur indirectly, for example through host-derived nucleic acids generated or released during prion-induced cellular stress or injury. These direct and indirect mechanisms are not mutually exclusive, but whether either contributes to activation of the IRF3–I-IFN pathway during prion infection remains unknown. Identifying the upstream signals that link PrPSc accumulation to IRF3–I-IFN activation will therefore be critical for understanding how prion propagation engages host innate immunity.
4. Which cell types mediate IRF3–I-IFN–Oas1a-dependent anti-prion responses in the CNS? Although neurons can mount innate immune responses, the relative contributions of neurons, microglia, astrocytes, and other CNS-resident cells to this pathway during prion infection remain unclear. Defining this cell-type specificity will be important for understanding both the protective and potentially detrimental effects of I-IFN signaling in the CNS.

## 10. Conclusions

Prion diseases represent a unique intersection of neurodegeneration and infectious pathology. Accumulating evidence indicates that innate immune signaling can influence prion propagation and disease progression in a context-dependent manner. Among these responses, experimental studies suggest that the IRF3–I-IFN–Oas1a axis can contribute to restricting prion propagation through mechanisms distinct from classical antiviral immunity. Oas1a, in particular, has been shown in experimental models to suppress PrP^Sc^ formation independently of RNase L through a noncanonical mechanism involving direct interaction with PrP^C^. However, the extent to which the IRF3–I-IFN–Oas1a axis is conserved across different prion strains and host species, particularly in human prion disease, remains to be established. Further investigation of its upstream activation and downstream mechanisms, together with studies in diverse prion models and human disease, will be essential for defining the biological and potential therapeutic significance of this emerging host response.

## Data Availability

No new data were created or analyzed in this study. Data sharing is not applicable to this article.
